# Contribution of the Gut Microbiota in P28GST-Mediated Anti-Inflammatory Effects: Experimental and Clinical Insights

**DOI:** 10.3390/cells8060577

**Published:** 2019-06-12

**Authors:** Benoît Foligné, Coline Plé, Marie Titécat, Arnaud Dendooven, Aurélien Pagny, Catherine Daniel, Elisabeth Singer, Muriel Pottier, Benjamin Bertin, Christel Neut, Dominique Deplanque, Laurent Dubuquoy, Pierre Desreumaux, Monique Capron, Annie Standaert

**Affiliations:** 1 Univ. Lille, Inserm, CHU Lille, U995—LIRIC—Lille Inflammation Research International Center, F-59000 Lille, France; benoit.foligne@univ-lille.fr (B.F.); marie.titecat@chru-lille.fr (M.T.); arnaud.dendooven@gmail.com (A.D.); pagny.aurelien@gmail.com (A.P.); elisabeth.singer@univ-lille.fr (E.S.); muriel.pottier@univ-lille.fr (M.P.); benjamin.bertin@univ-lille.fr (B.B.); christelneut@nordnet.fr (C.N.); laurent.dubuquoy@inserm.fr (L.D.); pdesreumaux@hotmail.com (P.D.); monique.capron@univ-lille.fr (M.C.); 2 Univ. Lille, CNRS, Inserm, CHU Lille, Institut Pasteur de Lille, U1019—UMR 8204—CIIL—Center for Infection and Immunity of Lille, F-59000 Lille, France; coline.ple@ibl.cnrs.fr (C.P.); catherine.daniel@ibl.cnrs.fr (C.D.); 3 Univ. Lille, Inserm, CHU Lille, CIC 1403, Centre D’Investigation Clinique, F-59000 Lille, France; Dominique.DEPLANQUE@CHRU-LILLE.FR; 4 Service des Maladies de L’Appareil Digestif et de la Nutrition, CHU Lille, F-59000 Lille, France

**Keywords:** helminth protein, immunization, inflammatory bowel diseases, fecal microbiota, fecal transplantation, mice, humans

## Abstract

An original immuno-regulatory strategy against inflammatory bowel diseases based on the use of 28 kDa glutathione S-transferase (P28GST), a unique schistosome protein, was recently proposed. Improvement of intestinal inflammation occurs through restoration of the immunological balance between pro-inflammatory T-helper 1 (Th1) responses and both T-helper 2 (Th2) and regulatory responses. However, detailed mechanisms explaining how P28GST prevents colitis and promotes gut homeostasis remain unknown. Considering the complex interplay between the adaptive and innate immune system and the intestinal microbiota, we raised the question of the possible role of the microbial ecosystem in the anti-inflammatory effects mediated by the helminth-derived P28GST protein. We first analyzed, by 16S rRNA sequencing, the bacterial profiles of mice fecal microbiota at several time points of the P28GST-immunomodulation period prior to trinitrobenzene sulfonic acid (TNBS)-colitis. The influence of gut microbiota in the P28GST-mediated anti-inflammatory effects was then assessed by fecal microbiota transplantation experiments from P28GST-immunized mice to either conventional or microbiota depleted naïve recipient mice. Finally, the experimental data were supplemented by the temporal fecal microbiota compositions of P28GST-treated Crohn’s disease patients from a pilot clinical study (NCT02281916). The P28GST administration slightly modulated the diversity and composition of mouse fecal microbiota while it significantly reduced experimental colitis in mice. Fecal microbiota transplantation experiments failed to restore the P28GST-induced anti-inflammatory effects. In Crohn’s disease patients, P28GST also induced slight changes in their overall fecal bacterial composition. Collectively, these results provide key elements in both the anti-inflammatory mechanisms and the safe therapeutic use of immunomodulation with such promising helminth-derived molecules.

## 1. Introduction

Over the last few decades, there has been increasing interest in the therapeutic potential of helminths in autoimmune disorders, such as multiple sclerosis, type-1 diabetes, rheumatoid arthritis, and inflammatory bowel diseases (IBDs) [1]. This has been assumed based on the “hygiene hypothesis” [2] and “old friend theory” [3]. Indeed, epidemiological reports described an inverse global incidence of allergic and autoimmune diseases rising in developed countries, whereas helminth infections were more prevalent in developing areas. Helminths are also known to be major modulators of the immune system since they characteristically induce T-helper 2 (Th2) and T-regulatory immune responses and reduce pro-inflammatory responses. Numerous animal studies and pre-clinical data have evidenced that helminth infections reduce the severity of inflammatory diseases and may protect the host against the development of autoimmune disorders [1]. As an alternative to the administration of whole living parasites, the current challenge is to promote the use of crude extract or, even better, purified or recombinant molecules derived from helminths, able to reproduce anti-inflammatory effects similar to those induced by the infection [4,5,6]. In this regard, we have demonstrated the beneficial effect of a recombinant schistosome–enzymatic protein, the 28 kDa glutathione S-transferase (P28GST), in prevention as well as in treatment of experimental colitis in rodents [7,8]. The P28GST immunization strategy used in the prevention of colitis was at least as efficient as an established *Schistosoma* infection to lower colitis symptoms both in rats and mice. We have shown that P28GST reduces colonic inflammation via reducing local expression of pro-inflammatory mediators (TNF-α, IL-17). This was associated with enhancing IL-5, IL-13, and IL-10 production together with eosinophil infiltration in the colon, shown to be essential to the protective effect of P28GST [7]. Given the promising preclinical effects, P28GST use, thus, represents a new immune-regulatory approach against IBDs. Following a Phase 1 trial [9], a Phase 2 clinical trial is currently in process to assess the safety of P28GST in patients with Crohn’s Disease (ClinicalTrials.gov, NCT02281916). 

The underlying mechanisms by which immunization with P28GST protects against colitis remain to be elucidated. P28GST combines the antigenic properties and enzymatic activities of anti-oxidant glutathion S-transferase (GST) [8] and prostaglandin D synthase (PGDS) involved in the anti-inflammatory PGD2 pathway [10]. Whereas systemic regulatory pathways of both innate and adaptive immune systems are involved [7], the impact of this modulation at the colonic level remains to be defined, for example, via possible changes in the nature of gut microbiota. This is an important consideration because gut microbiota is widely recognized to play a large part in the development of a mature immune system and in maintaining mucosal homeostasis. Moreover, disturbance of gut microbiota in terms of diversity and composition is directly implied in inflammatory bowel diseases, whereas modulation of commensal bacterial diversity (e.g., probiotics administration or selective fecal transplantation) has shown some beneficial effects on intestinal physiology. The effects of the microbiome on immunological players from epithelial cells and antigen-presenting cells to innate lymphoid cells and regulatory T cells involved diverse microbiota-derived bioactive molecules. Some of these molecules have already been identified for their effects on inflammation within the intestine and at distant sites. This has shifted from cataloging individual members of the commensal community to understanding their respective or collective contributions to the physiology of the host, which includes regulation of inflammation by microbiota interactions with the host [11]. Recent investigations have evidenced that helminth infection induced a shift in the intestinal flora that might contribute to the therapeutic effect of helminth therapy. The observed changes in microbial community composition and diversity are nevertheless depending on the helminth species, the type of infection (acute or chronic infections or long-term colonization) and the design of studies. Indeed, chronic infections with *Trichuris muris* or *Heligmosomoides polygyrus* result in a reduced diversity in microbial community composition (i.e., Bacteroidetes) with an increased abundance of the Lactobacillaceae family [11,12,13,14,15,16], in contrast to the study from Su et al. [17] in which *H. polygyrus* infection was accompanied with a marked increase in the abundance of Bacteroidetes, a decrease in Firmicutes and Lactobacillales and an exacerbated bacterial colitis [17]. Others only reported minor changes [18]. In a study using a primate model of idiopathic chronic diarrhea, *Trichuris trichuria* infection has shown to increase microbial diversity with the expansion of Bacteroidetes and Tenericutes and reduction of Cyanobacteria phylum [19]. In humans, the few investigations addressing the impact of helminth infection on gut microbiota revealed slight or no changes [20,21,22]. Although controversial, these studies suggest that changes to the gut microbiota should be considered when defining anti-colitic mechanisms of helminth-based therapy [23]. A relatively little-explored question is whether helminth products may impact the composition of the intestinal microbiota, which can influence the immunoregulatory and anti-inflammatory responses [24]. To our knowledge, there is, as yet, no study that demonstrates the impact of well-characterized helminth-derived molecules on gut microbiota, neither how much the microbiota could contribute to the anti-inflammatory properties of these molecules. Even so, one study suggested that ES-62, a secreted product by the filarial nematode *Acanthocheilonema viteae*, normalized the gut microbiome in protecting mice against collagen-induced arthritis [25]. However, immune responses, either natural or induced by treatment such as chemotherapy or anti-cancer immunotherapy, seem to rely on gut microbiota [26,27,28]. Some work has also evidenced that microbiota can influence vaccine responsiveness [29], while the possible impact of vaccination on gut microbiota is poorly reported. For all these reasons, we addressed the possible contribution of gut microbiota in the anti-inflammatory effects mediated by the schistosome-derived P28GST protein. Following our previous work [7], we first analyzed the overall composition of mice fecal microbiota at several time points of the P28GST immunization period, and found slight changes that were not sufficient to support the P28GST protective effect on colitis following microbiota transplantation experiments on naive animals. Lastly, we addressed the possible shift in the fecal microbiota of P28GST treated humans in a pilot clinical study, by temporal metagenomic analysis of fecal samples in Crohn’s disease patients. Together, our data suggested that gut microbiota is not essential for the beneficial effect of the P28GST, and confirms its therapeutic use in IBD patients.

## 2. Materials and Methods

### 2.1. Materials

Chemicals and reagents were purchased from Sigma–Aldrich Chemical (St Quentin Fallavier, France), unless otherwise stated. Batches of P28GST protein were produced and purified from recombinant *Saccharomyces cerevisiae* culture under good manufacturing practice conditions by Eurogentec S.A (Seraing, Belgium). P28GST was conserved lyophilized in NH_4_HCO_3_ 10 mM and 6% lactose. This preparation was re-suspended extemporaneously with or without aluminum hydroxide 0.2%, an adjuvant compatible with animal and human use (Alhydrogel®, Brenntag Nordig, Frederikssund, Denmark and Miltenyi Biotec, Paris, France).

### 2.2. Animal Experiments and Ethics Statements

BALB/c mice (female, 6 weeks of age) were purchased from Charles Rivers (L’Arbresle, France), and maintained in pathogen-free animal holding facilities. The animal experiments complied with French legislation (Government Act 87-848). All the studies were approved by the local investigational ethics review board (Nord-Pas-de-Calais CEEA N°75, Lille, France; protocol reference numbers 352012 and 19-2009R) and French government agreement n° APAFIS#7542-20 1608251651940).

### 2.3. Immunization Protocols

Immunization was performed in BALB/c mice by three subcutaneous injections (on the back between the shoulder blades) of P28GST at 5 µg.kg^−1^ with an interval of 2 weeks, as previously described [7]. Alternatively, control mice received either saline (CTL), adjuvant alone (Adjuvant) or P28GST with adjuvant (P28GST+ Adjuvant).

### 2.4. Induction of Trinitrobenzene Sulfonic Acid (TNBS) Colitis and Inflammation Scoring

The trinitrobenzene sulfonic acid (TNBS) model of chemically-induced acute colitis was implemented in 13-week-old female BALB/c (*n* = 10 mice per group), as previously described [30]. Anatomo-pathology and inflammatory read-outs including quantitative reverse-transcriptase polymerase chain reaction were reported elsewhere [31]. Briefly, 0.5 cm of median colon samples were homogenized using the FastPrep instrument (MP Biomedicals, Illkirch, France), total RNA was isolated using RNAspin columns (Macherey–Nagel, Hoerdt, France). Reverse transcription and real-time PCR were performed with reaction kits (the High-Capacity cDNA RT Kit, Applied Biosystems – Fischer Scientific, Illkirch, France) and reagents (Universal PCR Master Mix, Applied Biosystems – Fischer Scientific, Illkirch, France), according to the manufacturers’ instructions. Polymerase chain reactions (PCR) were performed with an MX3005P machine (Agilent Technologies, Les Ulis, France). A custom gene expression assay (TaqMan, Applied Biosystems) was used with commercially designed and validated primers, which are listed in Table S1 in the Supplementary Materials. The housekeeping gene, *beta actin*, was run as an internal control. Data were analyzed using the 2−ΔΔCt method and expressed as a fold-increase over the control group’s values.

### 2.5. Patients and Human Fecal Samples

Crohn’s disease (CD) patients were part of a phase 2a pilot clinical trial designed to assess the safety of P28GST, aiming to control inflammation in moderate CD (ClinicalTrials.gov, NCT02281916). Both the French Health Authority (Agence Nationale de Sécurité des Médicaments, ANSM) and the Nord-Ouest IV Ethics Committee approved the pilot study (EudraCT number 2013-000595-15) and all subjects gave informed consent to participate in the trial. Eight patients were included after intestinal resection surgery or in moderate CD. Patients enrolled received three subcutaneous injections (one injection per month) of 100 µg of P28GST associated with Alhydrogel® as recommended adjuvant. For this study, fecal samples prior to and after P28GST administration were only obtained for 5 patients and allowed microbiota analysis. The samples were frozen and stored at −80 °C until analysis.

### 2.6. Microbiota Analysis

The DNA extraction, 16S targeted metagenomic analysis, and bioinformatics analyses were achieved according to optimized and standardized methodologies developed by Genoscreen (Lille, France) and are fully detailed in the Supplementary Materials.

### 2.7. Antibiotic Treatment and Fecal Microbiota Transplant

In some experiments, naive aged-matched mice (*n* = 20) were treated with broad spectrum antibiotics for 5 days in order to facilitate further fecal microbiota transplant reconstitution (Abx) (Figure S4). The antibiotic cocktail composition and related procedure are provided in the Supplementary Materials.

Fecal samples of ten donor mice (200 to 300 mg each) were collected aseptically and pooled in 0.5% cysteine 1X PBS to maximize anaerobic bacteria conservation. The stools (100 mg.mL^−1^) from control or immunized donors were resuspended by vigorous mixture for 1 min and centrifuged at 2000 rpm for 2 min to pellet down debris. The supernatant was collected and delivered to the age-matched recipient mice via oral gavage (200 μL each recipient) within 15 min of excretion to prevent changes in bacterial composition. The recipient mice (*n* = 10 each group), either fed with normal diet and drinking water or treated with antibiotics were subjected to the microbiota transplant three consecutive days per week for three weeks, from the first P28GST immunization boost (see also Figure S4).

### 2.8. Statistical Analysis

All analyses were performed by comparing experimental groups with their respective controls in a non-parametric, one-way analysis of variance (the Mann–Whitney U-test) or a two-tailed Student’s *t*-test, as appropriate (version 6.0, GraphPad Software Inc., San Diego, CA, USA). Data are presented as the mean ± SEM. Differences were judged to be statistically significant when the *p* < 0.05. However, *p* between 0.05 and 0.1 are somewhere specified to indicate trends (*). * *p* < 0.05; ** *p* < 0.01; *** *p* < 0.001.

## 3. Results

### 3.1. P28GST Immunization Prevents Experimental Colitis in Mice

**Figure 1:**
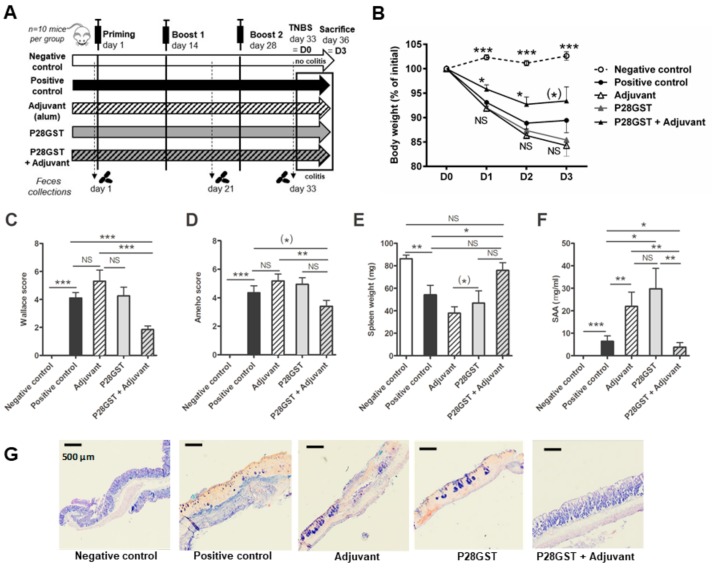
Prevention of 2, 4, 6-trinitrobenzene sulfonic acid (TNBS)-induced colitis in mice by 28 kDa glutathione S-transferase +Adjuvant (P28GST+Adjuvant) but not by adjuvant or P28GST alone. BALB/c mice received three subcutaneous injections of P28GST (5 µg.kg^−1^) without adjuvant, with adjuvant alone (aluminum hydroxide 0.2% solution), or P28GST combined with adjuvant, at 2 week intervals. Positive and negative colitis controls received saline. TNBS (100 mg.kg^−1^) was induced in all groups except negative control at day 33 and mice were sacrificed at day 36 (3 days after). Fecal samples were collected before the immune priming (day 1), 1 week after the first boost (day 21), and after the second boost (day 33). (**A**) Experimental design showing the immunization procedure, the fecal sampling plan and the final colitis schedule in all groups of BALB/c mice. (**B**) Percentage of loss of initial weight (prior colitis, at day 33 = D0) expressed as the comparison of individual weights recorded at killing (day 36 = D3). (**C**) Macroscopic clinical scores of colons at day 36 according to the scoring system of Wallace. (**D**) Histological Ameho’s scoring of colons at day 36. (**E**) Weight of spleens removed at day 36 expressed in milligrams (mg). Atrophy of spleens observed in positive colitis control, Adjuvant- and P28GST-treated animals was restored in the (P28GST+Adjuvant)-treated group. (**F**) Enzyme-linked immunosorbent assay was performed to quantify individual murine serum amyloid A protein (SAA) in all groups of mice. SAA levels expressed as mg per ml of blood serum were significantly lower in the (P28GST+Adjuvant)-treated group than others. (**G**) Representative images from May–Grünwald and Giemsa staining of paraffin-wax-embedded 6 µm sections of the colon of mice in all groups. Bars = 500 µm. Results are expressed as means ± SEM; *n* = 10 mice per group; * *p* < 0.05; ** *p* < 0.01; *** *p* < 0.001; ^(^*^)^ 0.05 < *p* < 0.1; NS, not significant is indicated when useful.

Before addressing the microbial basis of P28GST-induced anti-inflammatory effects, we first sought to confirm and extend the protection induced by the parasite protein P28GST in the acute TNBS colitis model in mice, as previously reported [7]. To this aim, we used a protocol consisting of three consecutive subcutaneous injections of either the adjuvant alone, as aluminum hydroxide, the recombinant P28GST alone, and the corresponding association, every two-weeks (see Figure 1A and method section). Respective groups of animals were then subjected to colitis one week following the last injection and sacrificed three days later to record inflammatory markers.

We confirmed the overall protective anti-inflammatory effects of P28GST protein when combined with the adjuvant, whereas P28GST or adjuvant separately did not. Indeed, while the TNBS-positive control group displayed a significant 15% body weight loss, the latter was lowered to below 10% by the P28GST+Adjuvant treatment, but not by the adjuvant alone neither with the sole protein (Figure 1B). In line, only the P28GST+Adjuvant treatment could significantly reduce the clinical macroscopic scores by more than 50%, *p* < 0.01 (Figure 1C), as well as the histopathological features observed in the positive control mice (Figure 1C,G). Compared with the negative control showing normal architecture of the epithelium, morphological appearance of tissue sections from the TNBS control and TNBS in adjuvant or P28GST alone showed extended epithelial lesions accompanied with necrotic areas and losses of the normal crypt structural organization. In addition, massive neutrophil infiltrates dominated the mucosa and the submucosa together with swelling and hemorrhagic zones. By contrast, P28GST+Adjuvant treated-mice had very less severe mucosa lesions and only moderate infiltrates and epithelial erosion were observed in the colons. Inflammation in the positive control group was associated with a near 40% decrease in the spleen weight when compared with healthy mice. The P28GST+Adjuvant partially recovered from this event of atrophy (Figure 1E). Similarly, and in contrast to other treatments, the high blood levels of serum amyloid A protein (SAA) related to the reference colitis were dramatically reduced by the P28GST+Adjuvant close to the baseline (Figure 1F).

**Figure 2:**
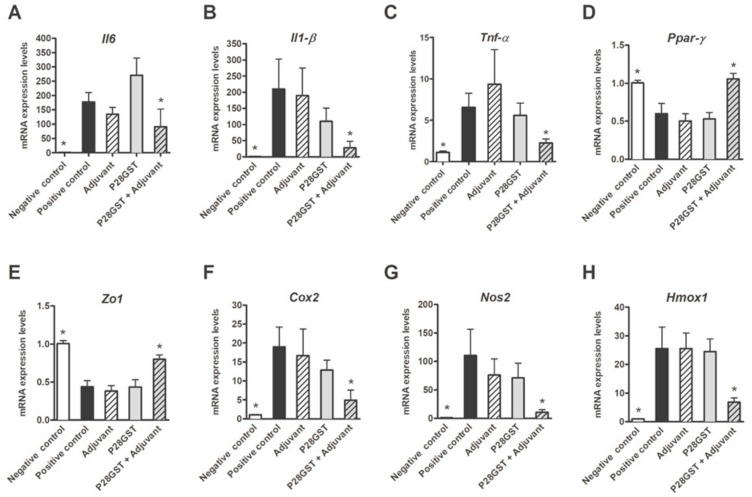
Mice receiving P28GST+Adjuvant exhibited a reduced inflammatory transcriptional signature when compared to the positive control, as well as in groups with adjuvant or P28GST alone. (**A**) Interleukin-6 (*IL6*), (**B**) interleukin *IL1-β*, (**C**) tumor necrosis factor alpha (*TNF-α*), (**D**) peroxisome proliferator activated receptor-γ (*Ppar-γ*), (**E**) Zonula occludens-1 (*Zo1*), (**F**) Cyclo-oxygenase-2 (*Cox-2*), (**G**) Nitric oxide synthase (*Nos-2*), and (**H**) Heme oxidase-1 (*Hmox1*) mRNA expression was assessed by quantitative PCR in the colons from the five groups of mice. Results are expressed as means ± SEM; *n* = 10 mice per group; * *p* < 0.05; NS, not significantly different from the positive control group.

Lastly, these observations were consistent with the transcriptional signatures in inflamed colons (Figure 2A–H). Indeed, the TNBS-induced upregulation of inflammatory genes (such as the immune-related *Il-6*, *Il-1β,* and *Tnf-α*) and oxidative-stress-related genes (such as *Nos2* and *Cox2*) was significantly less marked by the P28GST+Adjuvant treatment. The latter also normalized the drop in homeostatic genes which were downregulated in colitis (such as *Pparγ* and *Zo1*).

Both adjuvant and P28GST treatments, separately, had no beneficial effect on the abovementioned colitis markers. We, therefore, definitively confirmed that P28GST, in the presence of aluminum hydroxide, clearly alleviated colitis in mice by re-equilibrating the balance between pro-inflammatory and homeostatic genes.

### 3.2. P28GST Immunomodulation Slightly Modifies the Mouse Gut Microbiota Composition

Since the optimal conditions to reach a successful prevention of colitis were achieved in this study, we could address a posteriori the role of gut microbiota by the analysis of individual fecal samples (*n* = 8 per group) collected at distinct time points (D0, D21, and D33) before the onset of colitis (Figure 1A). Of note, beyond the comparison of microbiota composition from non-immunized control and P28GST+Adjuvant-immunized mice, metagenomic analyses in groups immunized with P28GST alone and adjuvant alone were also considered using 16S rRNA gene sequencing.

Rarefaction curves of bacterial species as a function of the number of sequences indicated the total bacteria diversity reached for the four experimental groups at the three considered time points (see Supplementary Materials Figure S1A–C). The average number of operational taxonomic units (OTUs) of 150 per individual is in agreement with other studies in mice [32,33]. At D0, before any treatment, the specific richness of observed OTUs was similar in all groups although the mean OTU number was slightly but significantly lower in the fecal samples from the adjuvant group. This may be due to the overall heterogeneity of laboratory mice. At D21, richness and Shannon indices were no longer different among groups, suggesting a stabilized diversity unaffected by housing conditions and treatments. However, at the D33 end point, unlike the alpha diversity, data were still comparable to the previous times, a slight decrease in diversity was only measured in the P28GST-treated animals (Supplementary Materials Figure S1D–I and S2C).

**Figure 3:**
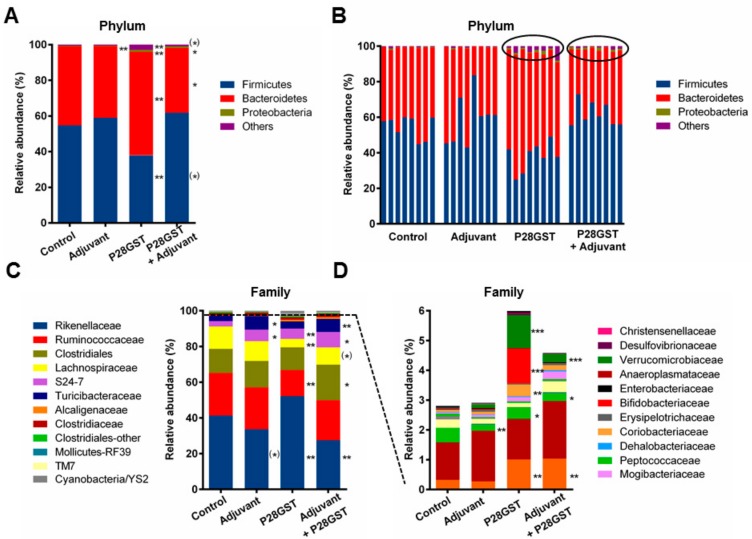
Immunizations of mice by Adjuvant, P28GST, and (P28GST+Adjuvant) induce slight changes in the composition of the fecal microbiota in comparison with saline as control. Data were generated by analysis of 16S rRNA gene sequences. (**A**) Mean relative abundance at the phylum level for each group of mice, *n* = 8 per group. (**B**) Individual relative abundance at the phylum level shows consistency of the effects. (**C**) Mean relative abundance at the family level for each group of mice and (**D**) focus on the corresponding low-represented mean relative abundance at the family level (below 6%), *n* = 8 per group. Results are expressed as % of the total operational taxonomic unit (OTU) numbers. * *p* < 0.05; ** *p* < 0.01; *** *p* < 0.001; ^(^*^)^ 0.05 < *p* < 0.1 versus the control group.

Concerning the composition of the microbiota at D33, the relative abundance of bacteria at the phylum level for the dominant groups such Firmicutes and Bacteriodetes was not modified by the treatment with adjuvant. The latter was weakly but significantly affected after treatment with P28GST either alone as well as with adjuvant (Figure 3A). The Firmicutes/Bacteroidetes ratio decreased in mice treated with P28GST alone but not in mice treated with P28GST+Adjuvant. Interestingly, we also observed an increase in the abundance of the minor phyla (less than 1%) representatives, i.e., Proteobacteria, and multiple other phyla for both P28GST-immunized groups, in the presence or absence of adjuvant. Of note, these observations were demonstrated for all individuals (Figure 3B). Likewise, bacterial communities at the family level were not strongly impacted by the various treatments, and although significant changes were observed, they mostly evoked weak variations of subdominant families already present in control mice (Figure 3C). For example, immunization by Adjuvant and P28GST+Adjuvant increased the proportion of Turicibacter and S24-7 bacteria and reduced Rikenellaceae. When looking at the minor species, some specific families which were very low or undetectable in controls were identified in P28GST alone (Bifidobacteriaceae, Coriobacteriaceae) or in the presence of adjuvant (Mogibacteriaceae, extended *TM7*) while other slight changes (extended Alcaligenaceae and Verrucomicrobiaceae) were shared by both P28GST alone and with adjuvant (Figure 3C,D). In fact, changes of microbiota composition with time, i.e., from D1 to D33 in control groups, were far more pronounced than those measured as a matter of immunomodulation treatment (Supplementary Materials Figure S3A,B).

Collectively, by using a metagenomic 16S sequencing approach, we assessed that the overall structure of the microbiota was relatively conserved with time and treatments. No massive shift of microbiota composition or dysbiotic states were induced by the distinct treatments. Although some obvious but negligible modifications in the bacterial community composition appeared with the various conditions, no single family (nor genus and even species, data not shown) seemed to be strongly and consistently associated with the a posteriori alleviation of colitis in mice.

In addition, we used culture methods based on selective media and specific conditions to compare non-immunized and P28GST+Adjuvant-immunized colonic flora (data not shown). This approach allowed us to distinguish between luminal and mucosa-associated epithelial flora and is complementary to the molecular methods. Here again, we observed some trends towards an increase in the absolute quantity of *lactobacilli* and decrease in enterobacteria associated with the recombinant parasite protein. However, they were not anywhere consistent enough to suggest a substantial role in the prevention of inflammation. Although we could not firmly identify any bacterial OTUs linked to the anti-inflammatory events, we decided to proceed to fecal transplantation experiments in order to fully explore the microbiome function of immunized mice.

### 3.3. Fecal Transplantation Conditions Are Crucial to Address Microbiota Functionality

To determine whether resistance to colitis was associated to the murine gut microbiota, we used a fecal transplantation approach. Immunomodulation protocol consisted in one priming and two boosts of either saline buffer (Non-immunized) as a control and the aluminum hydroxide adjuvant-associated recombinant P28GST (referred as Immunized) by subcutaneous injections as previously described. These mice respectively served as feces donors for non-immunized and P28GST-immunized naive recipients (Non-immu-R and Immu-R). Furthermore, two additional groups of naive mice, treated 5 days with a broad-spectrum antibiotic cocktail prior to the first transplant were used in order to facilitate the further colonization (Abx-non-immu-R and Abx-Immu-R). Three series of three fecal transplants were, thus, regularly achieved by freshly pooled feces from the immune boost (day 21) until the day of colitis (day 36) (Figure S4 and method Section 2)

**Figure 4:**
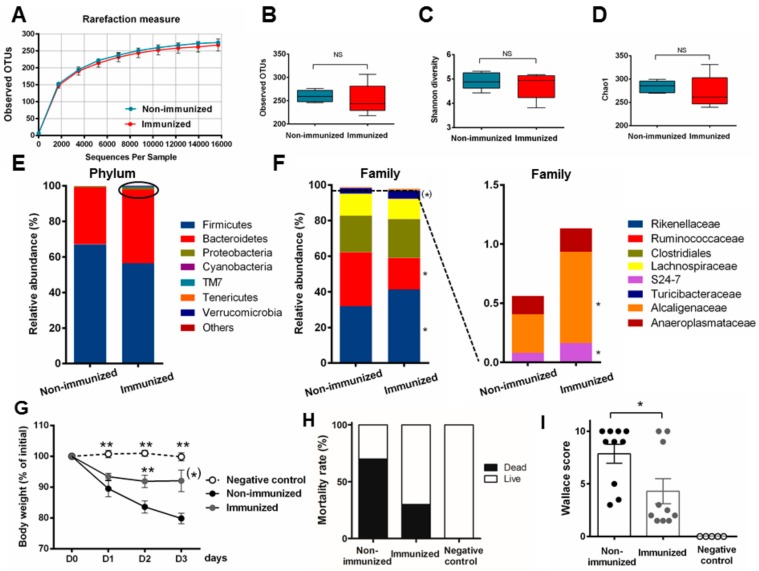
Immunization of BALB/c mice by P28GST+Adjuvant in another set of experiments reproduced subtle changes in fecal microbiota and consistently prevented inflammation in a more severe 2, 4, 6-trinitrobenzene sulfonic acid (TNBS)-induced colitis. (**A**) Rarefaction curves of observed operational taxonomic units (OTUs) as a function of reads show a similar number of sequences in the fecal samples of control (non-immunized) and immunized group of mice. Boxes and whiskers represent ± min/max of observed OTUs (**B**) Shannon index (**C**) and Chao 1 (**D**), respectively, showed no greater differences in alpha diversity and richness. Mean relative abundance at the phylum (**E**) and family level (**F**) with a focus on the low-represented OTUs at the family level (below 2%) for each group of mice. Results are expressed in % of the total OTU number, *n* = 5 per group for microbiota analysis (**A**–**F**). (**G**) Percentage of loss of initial weight (prior colitis, at day 36 = D0) expressed as the comparison of individual weights recorded at killing (day 39 = D3) as means ± SEM. (**H**) Mortality rate recorded at day 39, expressed in % of initial mouse number and (**I**) macroscopic clinical (Wallace) scores of colons at day 39 in both the non-immunized and immunized group of mice; means ± SEM. *n* = 10 per group for colitis evaluation parameters (**G**–**I**); * *p* < 0.05; ** *p* < 0.01; ^(^*^)^ 0.05 < *p* < 0.1.

In this second study, the impact of three injections by P28GST+Adjuvant on the fecal microbiota was also achieved. Of note, the microbial diversity in control mice was higher than in the first experiment, with an average of 250 OTUs. No differences in richness and OTUs numbers and alpha diversity were evidenced between non-immunized and immunized fecal samples (Figure 4A–D). Concerning the relative abundance at the phylum and family level, taxonomic distribution confirmed slight changes associated with immunomodulation. These variations, moderately distinct from those seen in the previous experiment, were still quite marginal. Again, some significant but marginal changes were seen on rare OTUs but P28GST+Adjuvant did not modify the general structure of the dominant bacterial phyla and families (Figure 4E,F). However, the group of immunized mice subjected to the TNBS colitis recapitulated a lower level of intestinal inflammation compared to non-immunized mice, as shown by a lesser amount of weight loss, reduced mortality, and a significant decrease in macroscopic inflammatory scores (*p* = 0.014), (Figure 4G–I). These non-immunized and P28GST-immunized healthy mice (Non-immu and Immu), respectively, serve as feces donors to transplant in naive recipients (Non-immu-R and Immu-R).

### 3.4. Fecal Transplantation in Naive Mice Is Not Sufficient to Restore the Phenotypic P28GST Induced Anti-Inflammatory Effects

**Figure 5:**
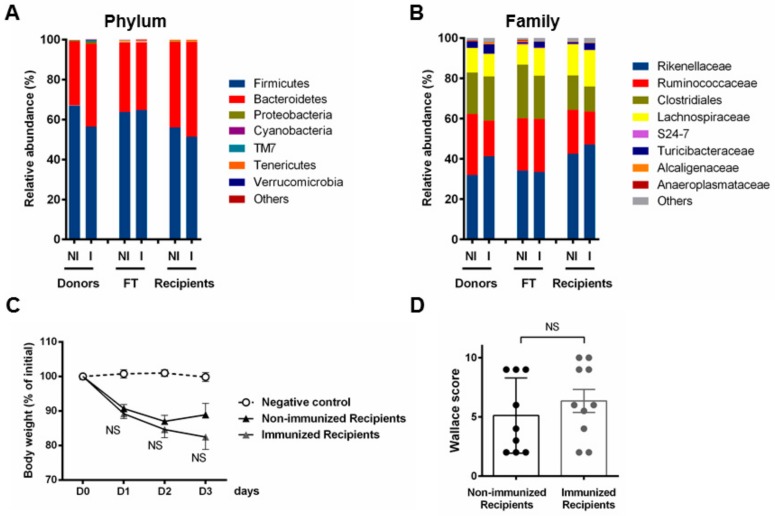
Fecal transplantation fails to induce substantial changes in microbiota and does not transfer resistance to colitis. Mean relative abundance at the phylum (**A**) and family level (**B**) for either non-immunized (NI) and immunized (I) feces of donor mice, *n* = 5 per group; the respective representative pooled fecal transplants (FT) (after resuspension, *n* = 1) and the corresponding feces of recipient mice, at day 36, *n* = 5 per group. Results are expressed as % of the total OTU number. (**C**) Percentage of loss of initial weight (prior colitis, at day 36 = D0) expressed as the comparison of individual weights recorded at killing (day 39 = D3) as means ± SEM, and (**D**) macroscopic clinical (Wallace) scores of colons at day 39 in both non-immunized and immunized recipient group of mice; means ± SEM. *n* = 10 per group for colitis evaluation parameters (**C** and **D**); NS, not significantly different from the positive control group.

To appraise the relevance of the bacterial contents to be transferred, we determined the microbial composition of the collected material (centrifuged supernatants of resuspended pooled fecal pellets) in comparison with the original structure of the microbiota of the donors. Bacterial compositions of the last fecal transplants on day 36 (FT-non-immu and FT-immu) were, respectively, very similar to the corresponding fecal content of the donors (Non-immu-R and Immu-R, *n* = 5 per group), both at the phylum and family level (Figure 5A,B). Fortunately, this suggests that the main detectable taxa occurring inside the gut were represented in oral preparations following the process. We only noted a slight decrease in the relative abundance of *Turicibacter* spp. but none or few OTUS were lost. Here, in contrast with previous results showing immunization-driven anti-inflammatory effects, no impact was recorded with respect to either body weight loss or the macroscopic scores following fecal transplantation between naive non-immunized and immunized recipient mice (Figure 5C,D).

### 3.5. Fecal Transplantation in Mice Treated with Antibiotics is Not Sufficient to Restore the P28GST Induced Beneficial Anti-Inflammatory Effects

**Figure 6:**
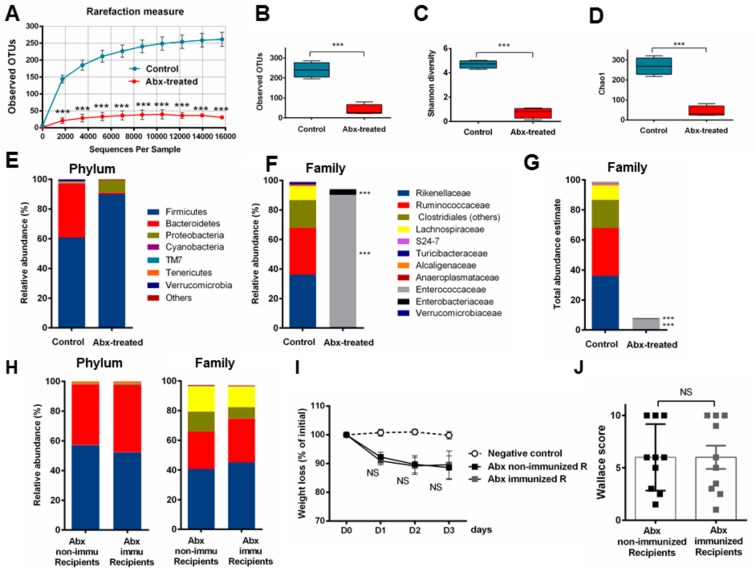
Antibiotic treatment profoundly changed the fecal microbiota to facilitate further recolonization but did not improve the resistance to colitis following fecal transplantation from P28GST-treated mice. (**A**) Rarefaction curves of observed OTUs as a function of reads showed a huge decrease of sequence numbers in the fecal samples of 5 day antibiotic-treated mice (Abx-treated) compared to the group of control mice. Boxes and whiskers represent ± min/max of observed OTUs. (**B**) Shannon index (**C**) and Chao 1 (**D**) confirms the drop in richness and diversity, *n* = 4 per group. Mean relative abundances at the phylum (**E**) and family level (**F**) from 16S sequence data demonstrated the drastic reduction of diversity while DNA quantification in fecal pellets allowed us to estimate the lowering of total abundance of bacteria with antibiotics at the family level (**G**). (**H**) Mean relative abundance at the phylum and family level for antibiotic-treated (Abx) recipient mice following (pooled) fecal transplantation from either non-immunized or immunized mice at day 36, *n* = 5 per group. Results are expressed as % of the total OTU number. (**I**) Percentage of loss of initial weight (prior colitis, at day 36 = D0) expressed as the comparison of individual weights recorded at necropsy (day 39 = D3) as means ± SEM and (**D**) macroscopic clinical (Wallace) scores of colons at day 39 in both non-immunized and immunized recipient group of mice; means ± SEM. *n* = 10 per group for colitis evaluation parameters (**I**,**J**); *** *p* < 0.001; NS, not significantly different from the positive control group

We first evaluated the impact of broad-spectrum antibiotics on the fecal microbiota by metagenomic analysis (*n* = 4 per group). Although we could not fully eradicate the microbes inhabiting the gut, we measured that five consecutive days of oral treatment drastically reduced the overall bacterial load as bacterial fecal DNA was 10- to 12-fold less abundant. The alpha diversity was also significantly reduced as demonstrated by the 10-fold lower numbers of OTUs and values of the Shannon and Chao1 indices which were expressively diminished (Figure 6A–D). The microbiota composition at the phylum levels also showed marked changes in relative abundance with antibiotics, leading to an over representation of Firmicutes and Proteobacteria to a lesser extent, accompanied by quite a total depletion of Bacteroidetes (Figure 6E). Analysis at the family and genus levels indicated that nearly 90% of the fecal bacterial abundances of antibiotic-treated mice were *Enterococcus* spp. and 4% Enterobacteria genus *Escherichia*. Such massive prevalence of *Enterococcus* was confirmed by culture methods and further identification using MALDI-TOF/MS (data not shown). The dominant strict anaerobic Clostridiales (Firmicutes) represented by Ruminococcaceae, Lachnospiraceae and other order representatives, together with Rikenellaceae (Bacteroidetes) in the control mice were no longer detectable in samples from antibiotics cocktail treated animals (Figure 6F). Moreover, taking into account the reduced bacterial load, we could estimate the absolute drop in quantity and diversity (Figure 6G). Consequently, the efficiency of treatment with antibiotics in view to remove most bacteria was attested, likely to optimize the success of the further microbial transplant. Following the antibiotic-mediated dysbiosis and depletion of most bacteria, fecal transplantations with time allowed the recovery of either diversity, phylum, and family relative abundances similar to the microbiota of naive and control mice. Enterococci and enterobacteria were no longer detectable in post-antibiotic-treated animals. As expected, we still could not differentiate the microbial composition between Abx-non-immu-R and Abx-immu-R (Figure 6H). Lastly, although we have maximized the conditions for exogenous flora implantation, the clinical symptoms of colitis, as supported by weight loss and macroscopic scores, did not demonstrate any beneficial role of microbiota in the context of P28GST-immunomodulation (Figure 6I,J).

### 3.6. Human Fecal Microbiota Composition Following P28GST Immunomodulation

**Figure 7:**
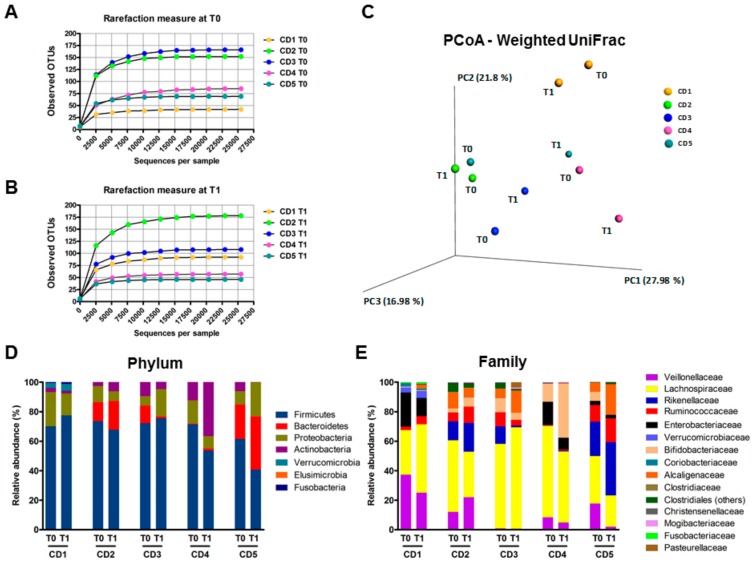
Fecal microbiota of Crohn’s disease patients was not modified following three subcutaneous injections with P28GST. (**A**) Rarefaction curves of observed OTUs as a function of reads demonstrated the great heterogeneity of microbiota among the patients at T0 and at T1 (**B**), *n* = 5. (**C**) Principal coordinate analysis (PCoA) of the fecal microbiota collected from 5 CD patients before and after P28GST injections using weighted UniFrac. Colors indicate samples from each individual. Comparison of beta diversity between before and after P28GST injections showed no significant difference with PERMANOVA test (*p* = 0.81, number of permutations 10,000). Individual relative abundances analyzed from 16S-based metagenomic sequences at the phylum (**D**) and at the family level (**E**) before (T0) and after achievement of the treatment protocol (T1) describe the well conserved distribution for each specimen.

In the framework of a Phase 2 clinical trial dedicated to safety assessment of P28GST immunomodulation in Crohn’s Disease (CD) patients, evaluation of gut microbiota before and after P28GST treatment has been planned as one of the secondary criteria. We were able to compare the composition of fecal microbiota in samples from five CD patients by Illumina 16S rRNA gene sequencing at baseline (T0) and after full achievement of the treatment protocol (T1), i.e., 3 months later from the first injection corresponding to 1 month after the last injection. As expected, the baseline fecal microbiota among individuals was rather heterogeneous, as illustrated by OTU numbers, ranging from 41 to 165 (Figure 7A) and the relative abundance of bacterial phyla and families at T0 (Figure 7D,E). Although the sole influence of the treatment at T1 is difficult to appraise in humans due to the several diet- and behavior-related biases that may interfere, the general structure of the microbiome was surprisingly well preserved. Indeed, both the phylum and family levels showed a quite stable representation of the main bacteria taxa with P28GST, with only marginal or no changes in the occurrence taxonomic groups. Indeed, the individuality was maintained and we could almost assign the T1 profile to the T0 based on similarity. Changes in human fecal microbiota of patients assessed after the treatment were in favor of an increase of richness and diversity. Indeed, three of the five patients had higher numbers of OTUs (Figure 7A,B) together with alpha diversity Chao1 and Shannon indices (data not shown). In accordance, the overall beta diversity (as determined by weighted UniFrac principal coordinate analysis PCoA) was no longer affected by the treatment in any of the patients (Figure 7C) and confirmed by no significant modifications of the Bray–Curtis dissimilarity index (data not shown). Although the purpose here was not to anticipate any changes in either beneficial or risk-associated bacteria, neither to correlate such changes with disease severity, the relative abundance of some specific taxa interestingly showed expansion endogenous of *bifidobacterial,* as presumable beneficial bacteria in three of the five specimens, together with lower amounts of proteobacteria, were more associated with deleterious contexts. Bacteria from the Veillonellaceae, known to be associated with pro-inflammatory events, also tended to decrease with the treatment (Figure 7D,E). Besides, due to the sample size of patients, we were not able to make a definite conclusion in terms of functionality and so the main consistent observation was that the immunomodulation procedure per se did not destabilize or exacerbate an already dysbiotic or imbalanced human microbiota.

## 4. Discussion

Here, we investigated the possible contribution of the gut microbiota in the anti-inflammatory effects of schistosome-derived P28GST. In line with our previous work [7], we confirmed the effectiveness of preventive subcutaneous injections with P28GST on experimental colitis alleviation in mice. We bring additional information that P28GST injections at a site distant from the bowel were able to lower further inflammatory symptoms in the colon. The present results clearly show that P28GST reduced the overall pro-Th1 cytokine immune response and restored the disrupted markers of mucosal barrier and oxidative stress. We also confirmed that such an immunomodulation approach requires the co-administration of alum as a necessary adjuvant. The mechanisms involved in helminth-mediated immunomodulatory properties are multiple, depending on the use of either the whole living parasite, crude extracts or purified or recombinant components [1,6]. The classical assumption indicates that helminth infections or helminth-derived molecules induce a type 2 immune response together with an immuno-regulatory response (including increase of IL-10, TGF-β, and CD4 + CD25 + FoxP3 + Treg cells) together with Th2-mediated tissue repair [34,35] that may counteract the Th1/Th17-mediated inflammatory effect observed in IBD. The dynamics of immune cells that orchestrate these effects remain unclear, and other complementary modes of action may also play a role, since dendritic cells [36], innate cells [37], and macrophages [38] are involved. Interestingly, the partial protection against colitis observed with helminth or helminth-derived molecules was restricted to post-inflammation intervention and no benefits were demonstrated with injection prior the colitis, suggesting downregulation of inflammatory events rather than preventive mechanisms.

In the present work, where immunization by a unique helminth protein (P28GST) alters the susceptibility of mice to colitis in a preventive manner, we previously demonstrated that such anti-inflammatory effects encompass the contribution of mucosal eosinophils, acting as T-helper-type (Th2) response inducers [7]. In addition to systemic and local induction of IL-13 and IL-5, a possible role of IL-25 can be hypothesized, as it has recently been shown in protection from amoebic colitis, acting via eosinophils and suppression of TNF-α through a new pathway of innate mucosal immune response [39].

As far as aluminum hydroxide has long been used to promote Th2 responses, a proposed mechanism is based on contribution of basophils [40], though other cell types can appear to be crucial. However, we cannot exclude that some extra phenomena could also contribute. Accordingly, in another context of a *Toxoplasma gondii* challenge, for example, it has been proven that immunization with TgHSP70, a heat shock protein, can induce protection and a massive anti-inflammatory event by strong oxidative effects at a distant site, despite no direct role on antibody neutralization nor control of the parasite infection processes [41]. A similar mechanism could occur also in favor of gut bacterial homeostasis and further prevention of colitis, as it was recently revealed [42], although it remains to be demonstrated. Collectively, the precise cellular mode of action induced by P28GST immunization remains to be elucidated, but preliminary results indicate the prominent role of regulatory cells (regulatory DC, T reg, and M2 macrophages) and anti-inflammatory IL-10 cytokine.

The possible role of the gut microbiota in this immunomodulation protocol is a key question that needed to be explored. In other words, does P28GST immunization modulate the gut microbiota? If so, are some of the protective effects supported by such bacterial shifts? In the present study, we clearly showed that the overall diversity and richness of mouse fecal microbiota was not influenced by P28GST immunomodulation. Only slight variations of very low-abundant taxa could be identified. To note, these marginal variations were moderately distinct between both our experiments, probably due to discrepancies in basal microflora from various animal batches and time, but without affecting the beneficial effects of the P28GST on colitis. This was strongly in contrast with a recent report showing that amelioration of allergic asthma followed by *H. polygyrus* infection involved the microbiota composition and subsequent short-chain fatty acids production to promote host regulatory T-cell responses [43].

Identification of modifications in the microbial community greatly depends on the methodologies used. Thus, a possible contribution of the gut microbiota on P28GST-mediated anti-inflammatory effects, if it exists, should be fairly subtle or involve another mechanism(s), such as an unidentified bacterium or a complex association of several microbes with immune-related and metabolomic-related consequences. However, our fecal transplantation approaches failed to demonstrate that gut microbiota modifications induced by P28GST+Adjuvant were able per se to restore the P28GST-induced anti-inflammatory effects. Considering the very close composition of the microbiota in donors and naïve mice, even in those pre-treated with antibiotics, it was, thus, not surprising that no differences in alleviation of colitis were observed after the fecal transplantation in both recipient groups, whatever their immunization status.

Notwithstanding, our results, are quite reassuring for safety reasons, since the stability of the intestinal microbial structure following P28GST immunization preserves gut homeostasis and its related immune and metabolic pathways. Interestingly, this is also valuable for the aluminum-based adjuvant alone, confirming, that adjuvant-dependent immunization is safe when considering gut microbiota and is not promoting dysbiotic states in contrast to emulsifiers [44] or specific diets [45]. Moreover, we also showed in clinical samples from CD patients receiving three injections of P28GST in the presence of adjuvant that the baseline microbiota of CD patients was unaffected by the treatment, whereas clinical scores and calprotectin levels decreased in parallel (manuscript in preparation). Studies on the composition of microbiota on suitable induction of protective immune response by vaccines are still in the early stage, and it is not clear how the gut microbiota affects the response to vaccination [29,46,47] and also how vaccination may in turn influence gut microbiota. Our results are, thus, in agreement with the data in the literature reporting that vaccination with bacterial antigens or virus does not alter or affect establishment of the intestinal microbiome, respectively, in mice and humans [48,49].

However, subtle or undetectable metagenomics changes in intestinal microbiota may have functional effects though, as it is the case for probiotic interventions. Consequently, the gut microbiota hypothesis cannot be ignored and we intend to test whether the fecal microbiota of vaccinated mice can nonetheless confer resistance to colitis. From a methodological consideration, addressing the role of gut microbiota in many diseases could be done by using negative conditions. This can be achieved either by lowering the intestinal bacterial load and inducing dysbiosis, e.g., in antibiotic-treated animals or even by fully suppressing the microbial part in axenic (germ-free) mice. This has been successfully used in several metabolic and neurologic disorders. However, it is well-known that gut microbes are necessary to induce and perpetuate the inflammatory processes. Many preclinical studies have shown that antibiotic therapy may attenuate colitis. Consequently, the disease outcomes are, thus, highly dependent on the microbiota after antibiotic treatment and using antibiotic treatments to address the role of microbiome is not appropriate here. In addition, antibiotic treatments only partially alter the intestinal microbiota and do not eliminate it. On the one hand, as we do not know which microbe(s) is/are putatively involved in the potential protection, we cannot discriminate whether the latter has been eliminated or not by the antibiotic cocktail. On the other hand, the release of microbial compounds following antibiotics may also induce local intestinal and further systemic strong immune responses that may interfere with colitis. Similarly, colitis cannot be strongly induced in axenic mice as microbiota is necessary to induce mucosal inflammation. Overall, several disadvantages of using axenic mice such as impaired immunological development would compromise immunization responses and colitis development. We, thus, excluded the use of germ-free mice in our experimental study. Of note, a recent study has investigated the anti-inflammatory role of a helminth-derived immunomodulator on the microbiome in an experimental mouse model of collagen-induced arthritis [25]. Interestingly, the protective effect against joint disease was reached using the subcutaneous administration route, likely in our model. The authors have reported that prophylactic depletion with broad spectrum antibiotics reduced the severity of arthritis while the compound acts to normalize the microbiome and maintain gut homeostasis. However, metagenomics was assessed in on-going arthritis and the normalization of microbiota observed was more reflective of the level of protection and reduced severity rather than the direct impact of the immunomodulator on the gut microbiota. To our knowledge, very few studies have yet investigated the direct impact of immunotherapy on gut microbiota, as has recently been done for pharmaceutical drugs [50].

Fecal transplantation is useful to study microbiota functionality and has been successful in many studies [51,52,53,54]. Use of axenic animals is no longer appropriate here, as a mature and functional immune system is necessary to evaluate susceptibility/resistance to infection and inflammation. We, thus, used a series of fresh fecal transplantations in both conventional and antibiotic-treated conventional recipient mice and neither strategy transferred any capacity to prevent colitis. Provided that all exogenous bacteria were effectively transferred to reach recipient mice intestines in sufficient amounts, these results emphasized that microbiota may not contribute to the P28GST-mediated protection of acute colitis. Indeed, we cannot completely exclude that some of the minor and very subtle microbial modifications could even influence some of the anti-inflammatory effects. Considering this hypothesis, the fecal transplantation method cannot allow to fully transfer all the microorganisms and overpass the original microbiota. We used antibiotic treatments to maximize acceptance of the exogenous flora by recipient mice and did three serial transplants according to the time to ensure an optimal imprinting. However, we cannot ignore that some key (and unidentified) bacteria were not able to reach the colon in a viable way. This can be due to the oxygen intolerance in the case of anaerobia bacteria (although we worked as fast as possible and supplemented the transfer buffer by cysteine to reduce the medium in order to lower this event). Another explanation is that some freshly collected colonic bacteria did not survive the upper gastrointestinal tract conditions, due to the gastric acidic environment, digestive enzymes, and further bile salt aggression.

Collectively, we concluded that there was (i) a low impact of P28GST immunomodulation on the gut microbiota and (ii) a lack of a major role for such microbiota in mediating protective effects. This is of major interest in the therapeutic perspective of P28GST in IBD patients, lowering the risk of antagonism due to the antibiotics or other antimicrobial treatments. Moreover, although the intrinsic heterogeneity of the gut microbiome in diverse human populations can orientate innate and adaptive immune responses [29], the fact that the microbiota was not the mediator of P28GST-based beneficial effects leads us to propose such treatment to patients, even though they are subject to dysbiosis and microbial imbalance, which could diminish immunogenicity in IBD. This is encouraging while we anticipate further promising results of further clinical trials aiming to target inflammatory bowel diseases. Moreover, our work also provides perspectives for the use of P28GST in other immune-related pathologies.

## Supplementary Materials

The supplementary materials are available online at https://www.mdpi.com/2073-4409/8/6/577/s1 [55,56,57].
