# Supplementary material for: Contribution of the Gut Microbiota in P28GST-Mediated Anti-Inflammatory Effects: Experimental and Clinical Insights
Source: Cells. 2019 Jun 12;8(6):577. doi: 10.3390/cells8060577 (PMC6627314; doi:10.3390/cells8060577)
Supplement: Supplementary file 1 [file cells-08-00577-s001.pdf]

cells MDPI

*Supplementary Materials*

## Supplementary Methods

### Antibiotic treatment and fecal microbiota transplant

In some experiments, naive aged-matched mice (n=20) were treated with broad spectrum antibiotics for 5 days in order to facilitate further fecal microbiota transplant reconstitution (Abx). The antibiotic cocktail was diluted in drinking water *ad libitum* providing a mouse may ingest approximately 2.5 ml per day. It consisted of amoxicillin:clavulanic acid at 1:0.2 ratio (1 g l-1, Sandoz), vancomycin (500 mg l-1, Mylan), metronidazole (1 g l-1, B. Braun), ciprofloxacin (200 mg l-1, Kabi). Taking the stability of antibiotics into account, a fresh mixture was reconstituted every 2-days and drinking bottles were protected from the light to avoid fluoroquinolone (ciprofloxacin) degradation. In addition, due to weak stability at room temperature, mice were orally fed daily with a freshly made solution of imipenem:cilastatin at 1:1 (250 mg l-1, Mylan). Control mice received standard drinking water by drinking bottle and gavage.

### Microbiota Analysis

#### *DNA Extraction*

DNA extraction was carried out on 200 mg of mouse or human feces according to an optimized and standardized protocol developed by the company Genoscreen, Lille, France. Based on the use of the QIAamp stool DNA Kit (Qiagen, France), various preliminary steps of mechanical and chemical lysis have been added in order to favor the lysis of all bacteria and in particular Gram +. A control of the extracted DNAs was performed with a fluorometric assay method for the SybrGreen Nucleic Acid I molecule (LifeSciences Merck, Germany) according to a protocol also developed by the company GenoScreen.

#### *16S targeted Metagenomic Analysis*

Microbial diversity and taxonomic composition of samples were determined for each sample using the so-called metagenomic targeted methodology. Briefly, a fragment of the 16S rRNA gene framing the V3 and V4 hypervariable regions was amplified from 5 ng of gDNA of the samples according to an optimized and standardized amplicon library preparation protocol Metabiote® (Genoscreen, France). The final amplification products (each containing a nucleotide index to differentiate the samples as well as the adapters necessary for carrying out the sequencing) were purified on beads and then finally mixed in equal concentrations. The sequencing was performed with the 2*250 bp chemistry on the Illumina MiSeq platform (Illumina, San Diego) at Genoscreen. The resulting raw sequences were subjected to a cleaning process comprising i) sorting the sequences according to the indexes and according to the 16S primers ii) the "trimming" of the sequences by truncating the bases from the 3 'end with a Phred threshold of 30 (iii) assembly the two paired sequences according to a minimum overlap of 30 bases and at least 97% identity over the overlapping area.

#### *Bioinformatics Analysis*

The computer analysis was carried out on a fully automated (Metabiote® OnLine) pipeline built around the QIIME v 1.9.1 software [55]. Following the steps of pre-processing, the full-length 16S rRNA gene sequences go through a step in which chimera sequences are detected and eliminated (in-house method based on the use of Usearch 6.1). Then, a clustering step is performed in order to group similar sequences with a nucleic identity defined threshold (97% identity for an affiliation at the genus level on the V3-V4 regions of the 16S rRNA gene) with Uclust v1.2.22q [56] through an open-reference OTU picking process and complete-linkage method, finally creating groups of sequences or "Operationnal Taxonomic Units" (OTUs). The most abundant sequence of each OTUs is therefore considered as the reference sequence of its OTU and is then taxonomically compared to a reference database (here Greengenes database, release 13_8; www.greengenes.gov) by the RDP classifier method v2.2 [57]. A final table listing all the created OTUs was generated comprising the number of sequences for each sample related to each OTU and their respective taxonomic affiliation. Alpha diversity indexes (Observed OTUs, Chao1 and Shannon) were generated from this OTU table.

## *Supplementary Tables and Figures*

### Supplementary Tables

**Supplemental Table S1:** list of targeted genes and corresponding primers accession numbers.

| Gene name | Abbreviation | Commercial reference |
|---|---|---|
| Actin beta | *ActB* | Mm 01205647_g1 |
| Nitric oxide synthase 2 (inducible) | *Nos2* | Mm 00440502_m1 |
| Heme oxidase 1 | *Hmox1* | Mm 00516004_m1 |
| Tumor necrosis factor | *Tnf* | Mm 00443258_m1 |
| Interleukin 1 beta | *Il-1b* | Mm 01336189_m1 |
| Interleukin 6 | *Il-6* | Mm 99999064_m1 |
| Prostaglandin synthase 2 | *Ptgs2* (*Cox2*) | Mm 00478374_m1 |
| Tight junction protein 1 | *Zo1* | Mm 00493699_m1 |
| Peroxisome proliferator activated receptor gamma | *Pparg* | Mm 01184322_m1 |

## Supplementary Figures

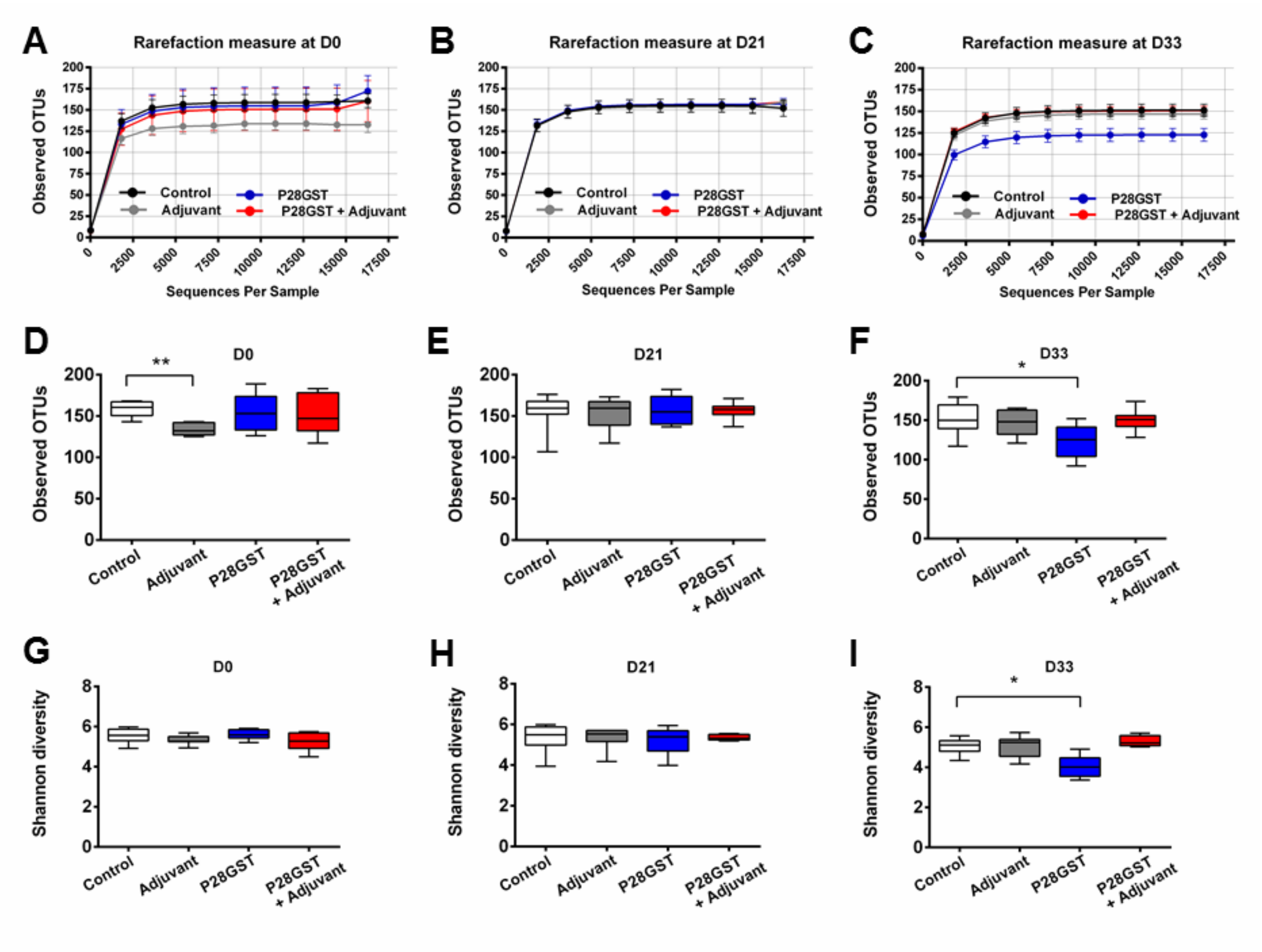


**Supplementary Figure S1.** Time course immunization by P28GST, adjuvant and their combination does not induce major changes in the overall richness and diversity of bacterial representatives in fecal microbiota. Rarefaction measure of observed operational taxonomic units (OTUs) are expressed as the number of OTUs as functions of sequences obtained per sample at D0 (A), D21 (B) and D33 (C). Total observed OTUs for each group are given at D0 (D), D21 (E) and D33 (F). The respective calculated Shannon diversity indexes are provided at D0 (G), D21 (H) and D33 (I). Data are represented as means ± SEM or ± min, max in box and whiskers representation; n=8 per group. *$P<0.05$; **$P<0.01$; ***$P<0.001$; (*)$0.05<P<0.1$.

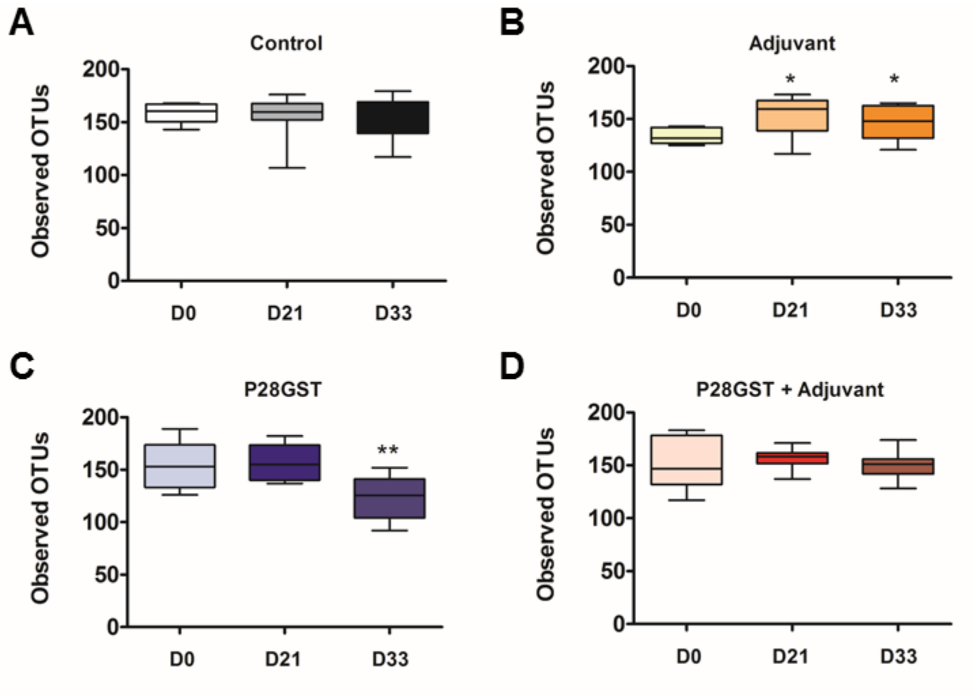


**Supplementary Figure S2**: Appraisal of immunization-induced richness in fecal microbiota overtime shows slight impact of treatments. No effect is observed in control mice (A), moderate changes in adjuvant- (B) and P28GST- alone (C) while vaccination by the combination do not modify the number of observed OTUs lastly (D). Data are represented as means ± min, max; n=8 per group. *$P$<0.05; **$P$<0.01; ***$P$<0.001; (*)0.05<$P$<0.1.

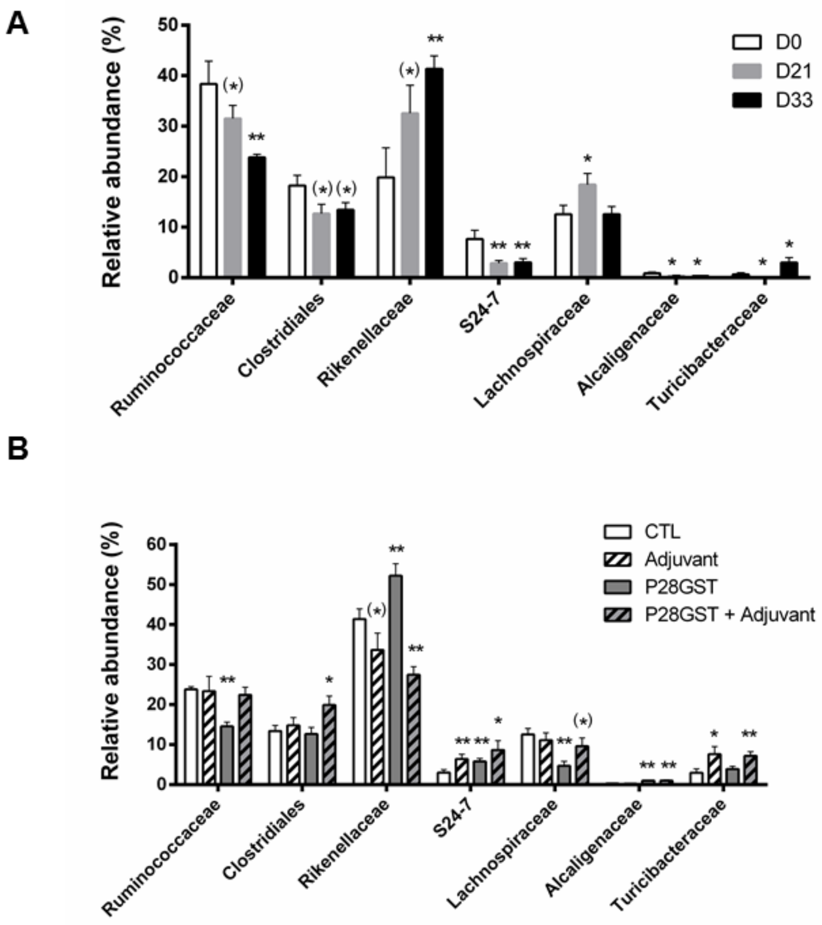


**Supplementary Figure S3:** Changes in the top 7 most abundant family taxa over time in the fecal microbiota of control mice illustrate the basal intragroup shift along with the 5 weeks housing period. Taxonomic profiles at the family level were analyzed by 16S rRNA gene sequencing at D0, D21 and D33 (A). In line, the magnitude of variations in the relative abundance of families with the distinct conditions at day 33 demonstrates significant but quite minor differences (B). n=8 per group. *$P$<0.05; **$P$<0.01; ***$P$<0.001; (*)0.05<$P$<0.1.

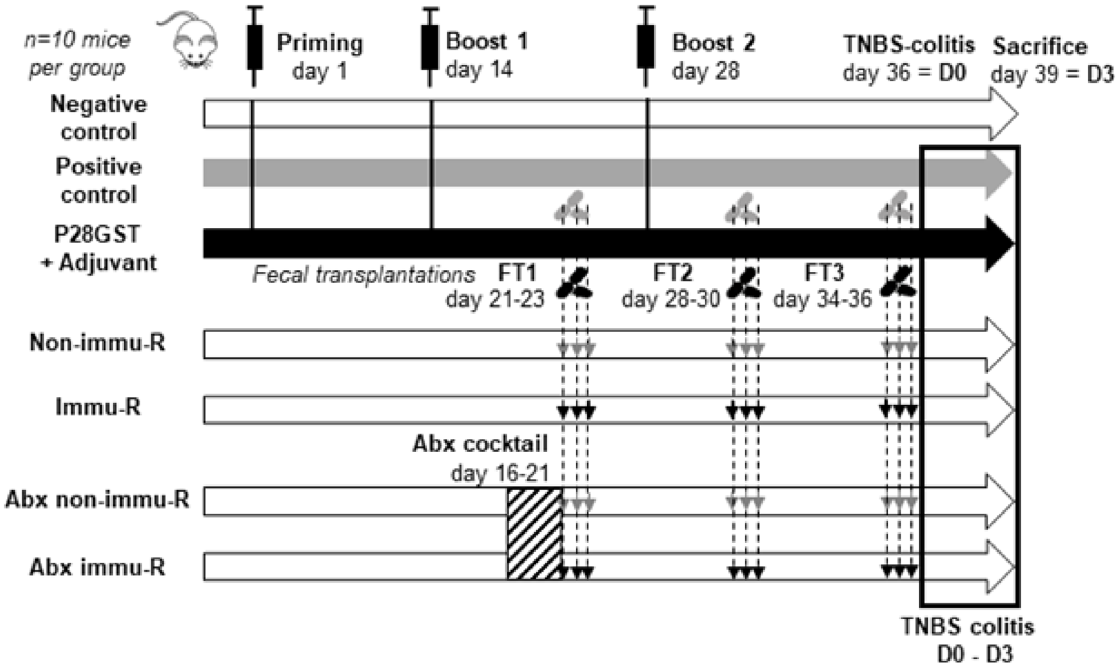


**Supplementary Figure S4:** Experimental design illustrates the immunomodulation procedure, the timing of fecal collections and the corresponding fecal transplantations (FT) before the final 3-days colitis in BALB/c mice. Pooled feces of either saline treated- and (P28GST+Adjuvant)-immunized mice provide stool material respectively for oral transfer in naive recipient mice (Non-immu-R and Immu-R) and in five-days broad spectrum antibiotic cocktail (Abx)- treated mice (Abx non-immu-R and Abx immu-R), n=10 per group.

**Supplementary References:**

55. Caporaso, J.G.; Kuczynski, J.; Stombaugh, J.; Bittinger, K.; Bushman, F.D.; Costello, E.K.; Fierer, N.; Peña, A.G.; Goodrich, J.K.; Gordon, J.I.; et al. QIIME allows analysis of high-throughput community sequencing data. *Nat. Methods* **2010**, *7*, 335–336.
56. Edgar, R.C. Search and clustering orders of magnitude faster than BLAST. *Bioinformatics* **2010**, *26*, 2460–2461.
57. Wang, Q.; Garrity, G.M.; Tiedje, J.M.; Cole, J.R. Naive Bayesian classifier for rapid assignment of rRNA sequences into the new bacterial taxonomy. *Appl. Environ. Microbiol.* **2007**, *73*, 5261–5267.
